# The Contribution of Genetic and Epigenetic Factors: An Emerging Concept in the Assessment and Prognosis of Inflammatory Bowel Diseases

**DOI:** 10.3390/ijms25158420

**Published:** 2024-08-01

**Authors:** Horia Minea, Ana-Maria Singeap, Manuela Minea, Simona Juncu, Cristina Muzica, Catalin Victor Sfarti, Irina Girleanu, Stefan Chiriac, Ioana Diandra Miftode, Carol Stanciu, Anca Trifan

**Affiliations:** 1 Department of Gastroenterology, Grigore T. Popa University of Medicine and Pharmacy, 700115 Iasi, Romania; horia.minea@yahoo.com (H.M.); simona.juncu@yahoo.com (S.J.); cvsfarti@gmail.com (C.V.S.); gilda_iri25@yahoo.com (I.G.); stefannchiriac@yahoo.com (S.C.); stanciucarol@yahoo.com (C.S.); ancatrifan@yahoo.com (A.T.); 2 Institute of Gastroenterology and Hepatology, “St. Spiridon” University Hospital, 700111 Iasi, Romania; 3 Department of Microbiology, The National Institute of Public Health, 700464 Iasi, Romania; manuela.minea@insp.gov.ro; 4 Department of Radiology, Grigore T. Popa University of Medicine and Pharmacy, 700115 Iasi, Romania; diandramiftode98@yahoo.com; 5 Department of Radiology, “St. Spiridon” University Hospital, 700111 Iasi, Romania

**Keywords:** inflammatory bowel disease, genomics, epigenomics, precision medicine

## Abstract

Inflammatory bowel disease (IBD) represents heterogeneous and relapsing intestinal conditions with a severe impact on the quality of life of individuals and a continuously increasing prevalence. In recent years, the development of sequencing technology has provided new means of exploring the complex pathogenesis of IBD. An ideal solution is represented by the approach of precision medicine that investigates multiple cellular and molecular interactions, which are tools that perform a holistic, systematic, and impartial analysis of the genomic, transcriptomic, proteomic, metabolomic, and microbiomics sets. Hence, it has led to the orientation of current research towards the identification of new biomarkers that could be successfully used in the management of IBD patients. Multi-omics explores the dimension of variation in the characteristics of these diseases, offering the advantage of understanding the cellular and molecular mechanisms that affect intestinal homeostasis for a much better prediction of disease development and choice of treatment. This review focuses on the progress made in the field of prognostic and predictive biomarkers, highlighting the limitations, challenges, and also the opportunities associated with the application of genomics and epigenomics technologies in clinical practice.

## 1. Introduction

Crohn’s disease (CD) and ulcerative colitis (UC) are heterogenous and relapsing intestinal inflammatory conditions that exert a severe impact on the quality of life of individuals and whose prevalence registers a continuous rise in many regions of the globe [1]. Dysfunction of the immune system and the intestinal microbiome combined with the intervention of various environmental factors, in a patient with genetic susceptibility, represent the main elements with a recognized contribution to the occurrence of IBD. Despite the numerous studies carried out in the last decades, the pathogenesis of these diseases is incompletely elucidated [2,3,4].

The clinical evolution varies between patients due to differences related to the localization and extent of intestinal inflammatory lesions, to which is added either the presence of extra-digestive manifestations or the development of severe complications [5]. The delay in establishing the diagnosis postpones the possibility of early initiation of the treatment, which substantially influences the progression of the disease and affects the health status of the patients [6]. Moreover, it has been observed that a favorable response obtained after the initiation of the administration of the therapy does not guarantee their long-term effectiveness, and the absence of a correlation between the clinical phenotype and serum or fecal biomarkers currently used to evaluate mucosal healing limits the possibility of adapting the therapeutic scheme [7].

Recently, it has been reported that various treatment options are only effective in approximately 40–60% of individuals because of molecular mechanisms involved in the modulation of intestinal inflammation [7,8]. This resistance associated with different drugs requires novel strategies by personalizing clinical management according to the new targets recommended by STRIDE II, which were defined as standardized treatment objectives to provide benefits for the patient [9,10,11]. Precision medicine, a strategy defined in 2011, recommends considering, mainly, the complexity of the enteropathogenic mechanisms—including clinical, genetic, and environmental characteristics—to improve the selection of therapeutic decisions with individual specificity [12].

Hence, biomarkers serve as straightforward and accurate, minimally intrusive instruments that could promptly steer clinical judgments and offer predictive insights into disease progression, treatment response, and postoperative recurrence [13]. A priority endorsed by the CORE-IBD initiative is the utilization of biomarkers to assess disease progression, the risk of complications, and the likelihood of adverse events linked to drug administration [14].

On the other hand, considering the heterogeneity of the phenotypes accounting for significant variations in the behavior of these diseases, it is unlikely that the determination of a single biomarker generates results that could be reliable for all patients. Additionally, it is crucial to acknowledge that focusing solely on a molecular marker followed by the search for a significant association with a clinical parameter could be an inappropriate orientation because it does not consider the complex perspective of the pathogenesis of IBD. An ideal solution is represented by the approach of multi-omics that investigates multiple cellular and molecular interactions, which are tools that perform a holistic, systematic, and impartial analysis of the genomic, transcriptomic, proteomic, metabolomic, and microbiomic sets. Moreover, the concept integrates clinical data to allow the construction of a map with individual specificity, which is useful for understanding the pathogenesis of IBD [1,15,16].

**Figure 1:**
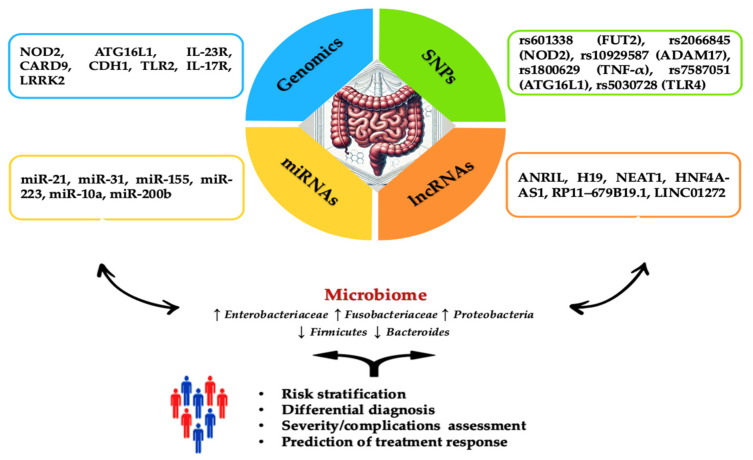
Overview of genetic and epigenetic factors for IBD assessment. The diagram summarizes the specific roles of key genes, SNPs, miRNAs, and lncRNAs involved in the incidence and progression of IBD. The blue section highlights genes such as NOD2, associated with an increased risk of Crohn’s Disease (CD) development. Moreover, it has a crucial role in recognizing bacterial components and modulating the immune response. ATG16L1 increases IL-1β production, intensifying inflammatory lesions in CD. IL-23R variants improve protection against IBD by regulating the Th17 cell-mediated immune response. CARD9 is involved in inhibiting intestinal inflammation through its role in signaling pathways associated with Toll-like receptors and C-type lectin receptors. CDH1 encodes E-cadherin, essential for maintaining the integrity of the intestinal epithelial barrier, and mutations in this gene compromise barrier function, contributing to IBD pathogenesis. TLR2 is involved in recognizing pathogens and initiating immune responses, playing a role in the expression of specific receptors and maintaining immune homeostasis in the intestine. IL-17R, the receptor for interleukin-17, is associated with various phenotypes ranging from risk to protection against IBD. LRRK2 modulates autophagy, a critical process for maintaining cellular homeostasis and immune response. The green section details SNPs like rs601338 (FUT2), which is linked to an increased risk of IBD in specific populations, such as Chinese. rs2066845 (NOD2) is connected to microbiome composition in relatives of CD patients while rs10929587 (ADAM17) increases the risk of surgery in CD patients. rs1800629 (TNF-α) is associated with higher production of TNF-α. rs7587051 (ATG16L1) is linked to decreased IFX levels in the GG genotype. rs5030728 (TLR4) is associated with decreased IFX levels in GG versus GA + AA. The yellow section emphasizes the importance of miRNAs, including miR-21, which stimulates the secretion of TNF-α and macrophage inflammatory protein 2 (MIP-2), intensifying inflammatory lesions. miR-31 is overexpressed in colonic epithelial cells specific to ulcerative colitis (UC), while miR-155 induces the differentiation of M1 macrophages. miR-223 inhibits NLRP3, reducing IL-1β release and promoting healing. miR-10a downregulates NOD2 and IL-12/IL-23p40 expression in inflamed mucosa, suppressing Th1 and Th17 cell responses. miR-200b inhibits TNF-α and IL-8, preventing increased paracellular permeability by redistributing tight junction proteins claudin-1 and zonulin-1. The orange section discusses the role of lncRNAs, such as ANRIL, which has low expression in intestinal mucosa correlated with CD susceptibility and differentiates between active and remission stages of the disease. High levels of ANRIL are associated with a favorable response to infliximab treatment. H19’s overexpression affects epithelial barrier integrity by reducing the release of miR-675, which encodes zonula occludens protein (ZO-1) and E-cadherin. Stimulated by IL-22, H19 inhibits negative regulators of intestinal epithelial cell proliferation and regeneration. NEAT1 inhibition reduces intestinal permeability and improves epithelial barrier integrity, mediating the innate immune response and the secretion of inflammatory cytokines. HNF4A-AS1 is related to the presence of mucosal ulcerations, with severe prognoses in pediatric patients. RP11–679B19.1 is a long non-coding RNA associated with the WWOX gene in patients with recurrent stenotic CD. It has been strongly linked to the risk allele A, indicating a significant association with fibrostenotic complications in CD. LINC01272 is overexpressed in patients with UC and CD, and its level is significantly correlated with severe mucosal ulcerations. **Abbreviations**: NOD2: nucleotide-binding oligomerization domain 2; ATG16L1: autophagy-related 16-like 1; IL-1β: interleukin-1 beta; CD: Crohn’s Disease; IL-23R: interleukin-23 receptor; Th17: T helper 17; CARD9: caspase recruitment domain family member 9; CDH1: cadherin 1; TLR2: toll-like receptor 2; IL-17R: interleukin-17 receptor; LRRK2: leucine-rich repeat kinase 2; SNP: single nucleotide polymorphism; FUT2: fucosyltransferase 2; ADAM17: ADAM metallopeptidase domain 17; TNF-α: tumor necrosis factor alpha; IFX: infliximab; TLR4: toll-like receptor 4; miR: microRNA; MIP-2: macrophage inflammatory protein 2; miR-31: microRNA-31; UC: ulcerative colitis; lncRNA: long non-coding RNA; ANRIL: antisense non-coding RNA in the INK4 locus; H19: H19, imprinted maternally expressed transcript; ZO-1: zonula occludens protein 1; IL-22: interleukin-22; NEAT1: nuclear paraspeckle assembly transcript 1; HNF4A: hepatocyte nuclear factor 4 alpha; HNF4A-AS1: HNF4A antisense RNA 1; RP11–679B19.1: long non-coding RNA RP11–679B19.1; LINC01272: long intergenic non-protein coding RNA 1272.

The inclusion of these techniques in the screening of the population is considered to be extremely promising for the detection of metabolic alterations in the early stage of the disease, being the support of future personalized medicine [17,18] (Figure 1).

In this review, we have assessed the progress made in recent years in the exploration of the genomic and epigenomic profile in the field of IBD, which led to the definition of promising biomarkers for the early diagnosis of complex interactions between molecular structures that have become therapeutic targets and objectives in clinical trials.

## 2. Genomics

### 2.1. The Involvement of Host Genetics in Susceptibility to IBD

The development of novel techniques led to the discovery of a significant number of genetic risk loci, characterized by a substantial heterogeneity of allele frequency and effect size in IBD, which outlined Mendelian traits within families, races, or populations [19,20]. Despite this progress, the literature provides a low rate of family history that varies between 1.5 and 28% in CD and 1.5 and 24% in UC [21]. It is estimated that hereditary factors have a stronger contribution to CD, with more than 120 genes identified, while only 22 genes have been defined for UC pathogenesis [22].

In first-degree relatives of these patients, the risk was estimated to be approximately twice as high for the development of CD (Relative Risk, RR: 7.77; 95% Confidence Interval, 95% CI: 7.05–8.56) compared to UC (RR: 4.08; 95% CI: 3.81–4.38) [23]. Moreover, it was found that the concordance rate for monozygotic and dizygotic twins reached up to 50% and 10% in CD, respectively, while in UC a lower heritability is reported (up to 15% and 4%) [24]. On the other hand, Spencer et al. reported numerous mutations in autophagy-related genes detected in the Ashkenazi Jewish population that were associated with an approximately four-fold higher prevalence of IBD and a frequent familial aggregation. However, by grouping these individuals according to the chronology of birth and age, the researchers emphasized the contribution of the common living environment that overlaps the genetic etiology for the onset of these diseases [25].

Considering these new perspectives, a genetic impact assessment has become an extremely interesting approach, both to predict IBD in the general population and perform a risk stratification, with better performance in CD (AUC = 0.75) than in UC (AUC = 0.70) [26]. Through sequencing studies, numerous independent coding variants have been identified in genes involved in modulating apoptosis and autophagy (NOD2, ATG16L1, IRGM, LRRK2) and inhibiting intestinal inflammation or infection (CARD9) [24,27]. The impact of genetic factors in modulating the expression of various receptors involved in the maintenance of intestinal barrier homeostasis is found in a broad spectrum of phenotypes ranging from risk to protection in UC and CD (Table 1).

Racial differences need to be considered when investigating genetic factors (Table 2). 

*NOD2 (CARD15)* is the gene associated with CD susceptibility [50]. The most common variants are R702W, G908R, and L1007fs. Patients with homozygous or compound heterozygous mutations have a 15–40-fold increased risk of CD development [54]. However, it has been observed that these alleles may occur in 0.5–2% of healthy individuals [24]. It was also found that the identification of a specific mutation (rs2066845) of this gene in first-degree relatives of patients with CD could explain the abundance of *Erysipelotrichaceae* in the feces. The results stress the hypothesis of the intervention of the genetic factor on the intestinal microbiome that occurs before the onset of the disease [54].

Further studies have highlighted the substantial contribution of *ATG16L1*, *IL-23R*, and fucosyltransferase 2 (*FUT2*) genes regarding autophagy, microbiota imbalance, and alteration of the epithelial barrier function in IBD that have been explored in different populations [44,55]. ATG16L1 deletion increases the production of IL-1β and causes the intensification of intestinal inflammatory lesions [53].

The major histocompatibility complex (HLA) genes, located on chromosome 6p21.3, encode specific proteins that initiate the T cell-mediated response and have a key role in most chronic inflammatory diseases [56]. Among these, the HLA-DRB1 and HLA-DQB1 alleles have the most consistent association with UC and CD, being involved in their severe evolution [57,58]. 

Gao et al. defined the risk for the development of UC depending on the presence of genes with a role in the regulation of the epithelial barrier (hepatocyte nuclear factor 4 alpha-HNF4A, laminin subunit beta 1-LAMB1, cadherin 1-CDH1, and G protein subunit alpha 12-GNA12), T cell proliferation and activation (TNFSF14), type I interferon expression (RFTN2), and Toll-like receptor stimulation (IRF5) [59]. The chromosome 1p31 is involved in the coding of a subunit of interleukin IL-23R, which has a determining role in the pathogenesis of intestinal inflammation. Loss of function of this receptor or alteration of cellular immunity associated with different IL23R variants improves protection against IBD [60]. Moreover, patients carrying different variants of TYK2 were also found to have protection from IBD through diminished T-lymphocyte response to IL-23 stimulation and reduced phosphorylation of STAT3 [61].

### 2.2. Interaction between Gut Microbiota and Genomics in the IBD Pathogenesis

An increased amount of evidence supports the role of genetic susceptibility on the homeostasis of the intestinal microbiota as an essential element for the triggering of an abnormal immune response and the alteration of the epithelial barrier [62]. Among the genes with impact in IBD, a significant association stands between Caspase recruitment domain family member 9 (CARD9), Nucleotide-binding oligomerization domain containing 2 (NOD2), Autophagy related 16 like 1 (ATG16L) and Fucosyltransferase 2 (FUT2), and the low abundance of the protective *Roseburia* species that switches acetate into butyrate [63]. NOD2 represents the most relevant susceptibility gene for Crohn’s disease, frequently associated with the phenotype that develops strictures, ileal damage, and presents an increased risk of surgical intervention [64]. This gene encodes an intracellular receptor that recognizes muramyl dipeptide from the microbial peptidoglycan structure to synthesize an oligomer capable of activating a pro-inflammatory signaling cascade and to stimulate autophagy through ATG16L1 [65]. Up to this moment, both experimental models in mice and studies in humans have emphasized the impact of these genes on the diversity of the microbiota. For example, in the intestinal mucosa of NOD2 knock-out mice, an increased abundance of microorganisms from the genus *Firmicutes*, *Bacteroides*, and *Bacillus* was found [66].

The establishment of intestinal dysbiosis alters the epithelial barrier by disrupting the tight junction proteins with a much more serious evolution of colitis compared to control groups of mice [67,68]. In CD patients, the polymorphism of these genes alters the ability of Paneth cells to recognize and eliminate pathogens, favoring the accumulation of the *Actinobacteria* group and the *Firmicutes/Bacillus* class, and a reduced presence of *Ruminococcaceae* family, which increases the potential for the development of intestinal inflammatory lesions [69].

The identification of a specific mutation (rs2066845) of NOD2 in first-degree relatives of patients with Crohn’s disease explained the abundance of *Erysipelotrichaceae* in the feces. These results stress the hypothesis of the intervention of the genetic factor on the change of the intestinal microbiome that occurs long before the onset of the disease [54]. Another gene involved in the recognition of pathogens is the caspase recruitment domain-containing protein 9 (CARD9) and causes a reduction in the response to immunoglobulin G in individuals with susceptibility loci for both CD and UC. Furthermore, biallelic loss-of-function (LOF) variants of CARD9 have been observed to be associated with increased susceptibility to *Candida albicans* infections [65]. Although LOF variants of the interleukin (IL)-23 receptor (IL23R) have been shown to protect against CD development, they increase susceptibility to various microbial infections. As a result, the blockade of IL12/23p40 and L23p19 through the administration of ustekinumab and risankizumab, respectively, has therapeutic efficacy in IBD [70].

### 2.3. Prediction of the Evolution of the Disease and Treatment Response 

The investigation of the genetic background has become an important element for an individualized therapeutic strategy based on optimizing the dose to which a favorable response is obtained, without triggering side effects [71]. Although many medications are effective, a frequently encountered problem in clinical practice is the loss of long-term response. In this context, a genetic risk assessment is an attractive strategy, both to predict IBD in the general population and to stratify patients according to their response to the chosen treatment [72,73].

A significant number of patients face an unpredictable evolution that complicates the decision to select medicines. Currently, a reduced number of genetic tests could anticipate the effectiveness of the response or the probability of developing toxic reactions [24,74,75]. Even though they have distinct pharmacodynamic profiles, the administration of anti-TNF monoclonal antibodies (infliximab, adalimumab, and golimumab) act identically, reducing the extent of inflammatory lesions and the probability of relapses, but approximately one-third of patients become non-responders, with genetic factors being responsible for this evolution [76].

Verstockt et al. observed that the measurement of a low signal for triggering Receptor Expressed on Myeloid cells 1 (TREM1) at the beginning of anti-TNF therapy is predictive of a favorable response associated with endoscopic remission, with better accuracy for mucosal expression (AUC = 0.77, 95% CI: 0.616–0.919; *p* = 0.003) than serum expression (AUC = 0.58, *p* = 0.31) [77]. A multigenic signature, different from the original profile of T lymphocytes, was defined by Biasci et al. to characterize an aggressive phenotype that presents an increased risk for treatment escalation in UC (Hazard Ratio, HR: 3.1; CI 95%: 1.25–7.72, *p* = 0.02) and CD (HR: 2.7, CI 95%: 1.32–5.34, *p* = 0.01). In this study, the sensitivity and specificity for predicting multiple exacerbations in the following 18 months of therapy was 72.7% and 73.2% in CD and 100% and 48% in UC, respectively [78]. In the same context, Lee et al. identified four different loci (FOXO3, XACT, IGFBP1, MHC) related to the susceptibility of treatment escalation in CD and, in addition, proved that rs7576459 associated with IGFBP1/IGFBP3 represents a clinical phenotype with a severe prognosis (Odds Ratio, OR: 3.91; 95% CI: 3.10–5.01; *p* < 0.001) [79]. Although pretreatment genotyping is recommended in the European Crohn’s and Colitis Organization (ECCO) guidelines, it has been proven that it could not replace the need to monitor the occurrence of possible adverse effects during the administration of certain drugs [80].

The HLA system is considered as a predictor of the occurrence of adverse effects during the administration of thiopurine, azathioprine, or mercaptopurine. Using the GWAS approach, two haplotypes were identified—HLA-DQA1*02:01 and HLA-DRB1*07:01—most often involved in the unpredictable evolution of therapy. Additionally, the risk of developing pancreatitis three months after the initiation of immunosuppressive therapy was 9% and 17% in heterozygous and homozygous patients, respectively, in direct relation to the presence of rs2647087 (OR: 2.59, CI 95%: 2.07–3.26, *p* < 0.001) [81]. Several studies have reported the involvement of single-nucleotide polymorphisms (SNPs) in the response to anti-TNF therapy in IBD patients (rs1800629, rs1799724, rs767455, rs1061624, and rs976881 with an inhibitory effect, while rs4149570, rs361525, and rs3397 determine a superior result), the majority being located in the TNF 1 (TNFR1) and TNF 2 (TNFR2) receptor genes and in those that regulate innate immunity (TLR4, IL6, IL1, IL17, TLR2, TLR9), autophagy, and apoptosis (Fas-L, CASP9, and ATG16L1) [82,83,84]. Genes that encode proteins involved in the regulation of the immune response have become a new target for evaluating anti-TNF treatment efficacy. Due to involvement in the pathogenesis of IBD, the genetic information for the synthesis of Toll-Like Receptor 4 (TLR4) possesses a role in the recognition of molecular patterns specific to certain pathogens, such as CD14 for ensuring homeostasis of the intestinal barrier [85].

Based on a retrospective evaluation in the PANTS study, it was observed in CD that baseline expression of major histocompatibility complex, antigen presentation, and myeloid cell enriched receptor were significantly higher in anti-TNF responders (infliximab and adalimumab) compared to patients who did not respond [46]. A model including 10 polymorphisms located in genes involved in the regulation of the NF-κB pathway (TLR2, TLR4, and NFKBIA) and signaling of TNF-α (TNFRSF1A) or other cytokines (NLRP3, IL1RN, IL18, and JAK2) was associated with the prediction of the rate of non-response to anti-TNF treatment, much higher in patients with UC (23%) compared to those with CD (13%) (OR: 1.98; 95% CI: 1.54–2.55, *p* = 0.0001). On the other hand, it was found that the inflammatory lesions genetically determined by the association of TNFRSF1A, IL1RN, and IL18 benefit from the favorable effect obtained by anti-TNF. Conversely, the intervention of IL-1β or IL-18 increases the probability of ineffectiveness as these cytokines could maintain inflammation even after the completion of therapy [86]. Recently, a significant number of studies have reported important correlations between different genetic polymorphisms and the response or lack of response to biological treatments, which reflected the heterogeneity of efficacy in IBD patients (Table 3).

Further studies concentrated on the genes involved in apoptosis and autophagy (BAX, BCL2, CASP3, and CASP9). It was observed that the low expression of these genes in peripheral blood lymphocytes influences the immune response, with the development of inflammatory lesions of the intestinal mucosa in UC. Moreover, the BAX/BCL2 ratio was significantly correlated (r = 0.473, *p* = 0.0095) with the duration of the active phase of the disease. Instead, in CD patients who received biological medication, a significantly lower (BAX/BCL2) ratio was noted compared to the placebo-treated group, suggesting that apoptosis modulation could become an important therapeutic mechanism in this pathology [97]. In various studies, numerous genetic polymorphisms that impact the alteration of cytokines (IL-1p, IL-1RA, IL-6, IL-11, IL-13, IL-17, and IL-27) were investigated. An example is the interleukin 17 (IL-17) family, a cytokine secreted by Th17 cells, which includes six ligands (IL-17A to IL-17F) and five receptors (IL-17RA to IL-17RE), located mainly in Paneth cells (IL-17A) and colonic epithelium (IL-17F). In CD patients, these receptors are overexpressed and transmit signals to downstream pathways through traf3-interacting protein 2 (TRAF3IP2), which share intracellular signal transduction molecules, such as I-κB and NF-κB, with the TNF-α signaling pathway. Hence, Urabe et al. managed to confirm the genotypes G/G for IL17F (rs766748) and C/C or C/A for TRAF3IP2 (rs1883136), which were strongly associated with a favorable response obtained after one year of treatment with IFX (OR = 37.92; *p* = 0.0019), with the sensitivity and specificity estimate of the genetic test being 70.0% and 100%, respectively [94].

In CD patients who received ADA, a pharmacogenetic relationship was highlighted between the polymorphisms rs755622 in the macrophage migration inhibitory factor gene (MIF) and rs3740691 in the ADP Ribosylation Factor GTPase Activating Protein 2 gene (ARFGAP2). However, the most consistent association with establishing a favorable response after 30 weeks of therapy was found for the variant rs2275913 in the IL17A gene (*p* = 9.73 × 10^−3^) [98]. It was observed that IL-23 cytokine, the ligand for IL23R, along with IL-12, to which it is related through the p40 subunit, became targets for the action of neutralizing antibodies in the treatment of IBD. However, it has been shown that the anti-inflammatory effect is the result of blocking the p19 subunit included in IL-23, with the generation of selective antibodies for therapy with guselkumab, risankizumab, and mirikizumab that act either by disrupting signaling pathways (JAK-STAT, NF-κB) or causing the loss of T helper-17, CD4+, and CD8+ cell function [99]. Although genetic risk assessment is considered a useful approach for the stratification of IBD patients, with increased performance in CD compared to UC, it has not yet been implemented in medical practice due to the increased costs of obtaining minor benefits [72].

## 3. The Role of Epigenetic Mechanisms on Prognosis and Therapeutic Response in IBD

The description of the dynamic and reversible changes that occur in the regulation of gene expression through interaction with various environmental factors, without altering the DNA sequence, represents a new direction of multi-omics research that has proven useful for understanding the pathogenesis of IBD in order to achieve precision medicine [24,84]. In recent years, evidence has appeared regarding the contribution of epigenetic mechanisms (hypo- or hypermethylation of DNA, histone modification, chromatin remodeling, or non-coding RNA sequences) for the maintenance of homeostasis of the intestinal epithelium, cell development, and differentiation or modulation of immune responses against pathogens [100]. By applying epigenome-wide association studies (EWAS), these signatures have been found to be stable over multiple cycles of cell division and replication with the possibility of being inherited [101]. Moreover, it was observed that the different epigenetic profiles could be used as biomarkers for early diagnosis and differentiation of clinical phenotypes; simultaneously, they became new therapeutic targets that could predict the response and occurrence of adverse reactions in IBD patients [100]. One of the epigenetic mechanisms that provides a new perspective on the pathogenesis of IBD is DNA methylation in gene promoters, with a key role in reducing their expression from a transcriptional point of view [102]. The methylation characteristics proved to be important for differentiating the types and activity stages of these diseases [100]. 

The detection of an increased number of differentially methylated positions (DMPs) in both pediatric and adult cohorts justifies its use for clinical phenotype classification. An analysis performed on rectal biopsy samples highlighted a methylation profile in the Thyroid Hormone Receptor Associated Protein (THRAP2), Forssman Glycosyltransferase 1 (GBGT1), Tumor Necrosis Factor Ligand (TNFSF), and Fucosyltransferase 7 (FUT7) that allows the differentiation of IBD patients from healthy controls [103]. Although these epigenetic features are significant, they disappear after mucosal inflammation is reduced by treatment. This finding suggests that abnormal DNA methylation might no longer exist when disease activity is reduced [104]. Furthermore, evidence supports that local inflammation could accelerate the methylation process, favoring the disruption of cellular replication and differentiation. As a result, DMPs could be used as markers for the early identification of tumors or dysplastic lesions occurring in patients with IBD [105,106].

An important role in regulating the structure or function of chromatin, with a major impact on the dynamics of various cellular processes, belongs to the post-translational modifications of histones (H2A, H2B, H3, H4, and H1) catalyzed by specific enzymes, such as histone-acetyltransferases (HAT), histone deacetylases (HDAC), histone methyltransferases (HMT), and histone lysine transferases (KAT) [107,108,109]. The progress registered in recent years regarding the exploration of the impact of chromatin changes has offered perspectives for the approach of new therapeutic interventions in these diseases (Table 4).

### 3.1. The Patterns of lncRNAs in IBD

Although they do not contain genetic information for protein synthesis, long non-coding RNAs (lncRNAs)—sequences made up of more than 200 nucleotides—have an important role in various biological processes by modulating the expression of genes involved in post-transcriptional/transcriptional regulation or by guiding chromatin-modifying complexes into specific genomic loci [128]. Recent cumulative data from GWAS have proven that lncRNAs promote inflammation and carcinogenesis, being associated with numerous diseases, which represents a promising perspective for clinical applications [129]. 

Even though their potential has not been sufficiently explored, it was discovered that they initiate and influence the evolution of inflammatory intestinal diseases, intervening in the maintenance of the integrity of the intestinal epithelium barrier, apoptosis, and the interaction of immune cells, which allowed their use as diagnostic and prognostic biomarkers [125]. Numerous studies have revealed the link between lncRNAs and the disruption of epithelial barrier function, reduction in junctional proteins, and increased intestinal permeability, which are predictive aspects for IBD relapse. LncRNA nuclear paraspeckle assembly transcript 1 (NEAT1) is a key component that mediates the innate immune response and the secretion of inflammatory cytokines. Liu et al. found that inhibition of NEAT1 expression in TNF-α and dextran sodium sulfate (DSS) reduced intestinal permeability and improved epithelial barrier integrity [130]. 

In various tissues during the embryonic stage, the lncRNA transcribed by the H19 gene on chromosome 11 is silenced after birth. In pathological conditions, the increased modulation of H19 induced by IL-22 inhibits the negative regulators of the proliferation and regeneration of intestinal epithelial cells (protein p53, miR-34a, and let-7), which stimulates mucosal healing [131]. However, overexpression of H19 was observed to reduce the release of miR-675 encoding zonula occludens protein (ZO-1) and the E-cadherin protein, strongly affecting epithelial barrier integrity [132].

#### 3.1.1. lncRNA as Prognostic and Diagnostic Biomarkers in IBD

Even though numerous studies have focused on the evaluation of the genomic impact, there is evidence that lncRNAs have an important role in the regulation of cellular physiology and are differentially expressed in IBD. The monitorization of the level of these lncRNAs could be applied to clinical evaluation and prognosis. As an example, the dysfunction or upregulation of lncRNAs (DIO3OS, KIF9-AS1, and LINC01272) is considered a biomarker that has an increased performance to differentiate IBD patients from healthy controls [125]. An important aspect refers to the possibility of using lncRNAs to investigate the late stages of the disease in which various complications are present (Table 5).

#### 3.1.2. lncRNAs as Predictors of Therapeutic Response in IBD

Several studies have concluded that certain lncRNAs might be considered excellent therapeutic targets. Among these, an anti-inflammatory small molecule drug (ABX464) stands out, a new antiviral drug with modulating potential for lncRNA 0599–205, which, in addition, triggers strong anti-inflammatory reactions in the dextran sulfate sodium (DSS)-induced colitis model [135]. 

New molecular therapies aimed at controlling intestinal permeability stimulate the increase in the level of lncRNA SPRY4-IT1, which has a protective effect on the epithelial barrier through the overexpression of tight junction proteins (claudin-1, occludin, and JAM-1) [136]. Additionally, ANRIL became a target that reflects the effectiveness of infliximab treatment for these patients. High concentrations were detected in responders compared to non-responders, who maintained a constant level of this drug [126].

Corticosteroids represent effective drugs to induce short-term emission in IBD. However, there are patients who develop resistance to this therapy due to the cytoplasmic accumulation of lncRNA (GAS5) that intervenes at the post-transcriptional level. As a result, GAS5 could be considered a biomarker with increased potential for personalizing corticosteroid therapy [137].

### 3.2. Role of miRNAs in IBD

In recent years, there has been increased interest in exploring the potential of a class of small molecules of endogenous origin, consisting of non-coding single-stranded RNA, with a length of 18–25 nucleotides (miRNAs), which have begun to be used as diagnostic and prognostic biomarkers of severe evolution or unfavorable therapeutic response in a variety of autoimmune, neurodegenerative, cardiovascular, or neoplastic diseases [138,139]. Moreover, it was discovered that in UC and CD, they maintain a key role in the post-transcriptional regulation of genes involved in the intestinal modulation of innate and adaptive immunity, in response to cellular changes or the action of environmental factors. This process affects autophagy, the homeostasis of the intestinal microbiome, and the integrity of the epithelial barrier [140].

In the context of complex pathogenesis, the modification of the expression profile of miRNAs due to various mechanisms, including DNA methylation, deacetylation, and keratin modification, acts on the intercellular signaling pathways, intervening in the synthesis of pro- or anti-inflammatory cytokines and regulating the permeability of the intestinal mucosa (Table 6).

#### 3.2.1. miRNAs as Diagnostic Biomarkers of IBD

Different types of miRNAs have been evaluated in numerous studies. A list of potential biomarkers for diagnostic and prognostic assessment in IBD patients is included in Table 7. After comparing the expression of miRNAs in tissues, feces, and peripheral blood, a repeated presence of miR-16, miR-21, miR-155, miR-223, or miR-31 is noted, which proved a functional relevance, both for patients with UC and CD, as well as in experimental models on mice [156]. A potential diagnostic marker for UC is represented by miR-21, with a significantly higher level compared to CD, which is mainly detected in inflamed lamina propria cells and partially damaged crypts [157]. It has also been shown that the increased serum level of this miRNA accompanied by an overexpression for miR-92a has excellent performance in differentiating UC from healthy subjects [158].

Other researchers have found that the upregulation of miR-31–3p in colonic epithelial cells is specific for UC. Furthermore, intracolonic administration of a chemical inhibitor of miR-31-3p to mice was reported to exacerbate DSS-induced colitis by stimulating the release of pro-inflammatory cytokines (IL-6, IL-8, and TNF-ά) [159]. Differences in miRNA composition and expression levels have also been suggested to be influenced by the evolution of the disease. As a result, it was found that miR-31 expression in sigmoid colon biopsies is strongly regulated in favor of the active UC form (up to 11 folds) compared to healthy controls [160]. In contrast, in CD patients, there was an upregulation in the inflamed duodenal mucosa for miR-146a and miR-155 [161]. 

On the other hand, it was found that the active form of this disease was associated with a much higher serum level for miR-155 compared to patients in remission [162]. Although differential diagnosis between CD and UC is considered a real challenge when inflammatory lesions are limited to the colon, a report indicated that a complex panel including miR-19a, miR-21, miR-31, miR-101, miR-146a, and miR-375, with an increased expression in saliva and blood, has a superior diagnostic performance [163].

**Table 7:** Correlation between IBD diagnosis and miRNAs.

miRNAs	Disease	Sample	Main Findings	References
**miR-21** **miR-126**	UC CD	Colonic tissue	Increased levels of miR-21 (6.7-fold) in lamina propria and miR-126 (2.3-fold) in endothelial cells in UC compared to controls; miR-21 is upregulated in UC compared to CD (5.8-fold).	[157]
**miR-31**	UC CD Controls	Colonic tissue	Hyperexpression in UC compared to CD and controls.	[159]
	UC controls	Colonic tissue	Increased expression in active form of UC (11-fold) and inactive form (3-fold) compared to healthy controls.	[160]
**miR-16, miR-21,** **miR-223, miR-155**	CD UC controls	Blood Fecal samples	Active CD was associated with higher miR-155 expression in blood samples compared to controls (1.9-fold) and subjects (2.1-fold) in remission (AUC = 0.752; 95% CI: 0.61–0.89).	[164]
**miR-146a, miR-155,** **miR-122**	CD UC controls	Colonic tissue	miR-146a and miR-155 were higher in the inflamed mucosa of children with CD and UC than in the intact mucosa. Elevated expression of miR-122 in CD compared with controls and UC.	[162]
	CD controls	Colonic tissue		[161]
**miR-125b, miR-223,** **miR-138, miR-155**	UC controls	Colonic tissue	Upregulation in inflamed UC tissues: miR-138 (10-fold), miR-223 (10-fold), miR-125b (2.56-fold), and miR-155 (2.33-fold).	[165]
**miR-141, miR-200a,** **miR-200b, miR-200c,** **miR-429**	UC CD	Colonic tissue	miR-141, miR-200b, and miR-429 were downregulated in CD and UC patients compared to healthy controls. miR-141, miR-200a, miR-200b, and miR-200c were significantly downregulated in CD in comparison to UC.	[166]
**miR-21-5p**	UC controls	Blood Colonic tissue	Downregulated in UC patients compared with control and determines inhibition of the expression of IL-6, TNF-α, IL6R, STAT3, ICAM-1, NF-κB, cleaved caspase-3, cleaved caspase-9, and FasL to alleviate the inflammation and apoptosis.	[167]
**miR-21** **miR-92a**	UC controls	Blood Colonic tissue	Overexpression has increased performance for differentiating UC from healthy subjects (AUC = 0.979, specificity = 1.00).	[158]
**miR-215-5p, miR-203a-3p, miR-223-3p, miR-194-5p, miR-192-5p, miR-10b-5p, miR-10a-5p, miR-337-5p, miR-582-5p**	CD controls	Colonic tissue	Downregulated in inactive CD compared to controls. Their inhibitory action on NOD2, TLR4, and IL6ST genes is involved in the innate immune response and cytokine signaling.	[168]
**miR-29b-3p, miR-122-5p, miR-146a-3p, miR-150-5p, miR-192-5p, miR-194-5p, miR-375-3p, miR-148a-3p, miR-199a-3p**	experimental model colitis mice	Blood	Hyperexpression of miR-29b-3p, -122-5p, -192-5p, -194-5p, -375-3p, -150-5p, -146a-3p, and the downregulated miR-148a-3p and -199a-3p; differentiation of UC patients from healthy controls (accuracies of 83.3%).	[169]
**mi146b-5p**	CD UC controls	Blood	The serum level (2.87- and 2.72-fold higher in patients with CD and UC than controls, respectively) is correlated with disease activity (CDEIS r = 0.579; UCIES r = 0.582) (AUC = 0.869, 95% CI: 0.764-0.940).	[170]

**CD**: Crohn disease, **UC**: ulcerative colitis, **CDEIS**: Crohn’s disease endoscopic index of severity, **CI**: Confidence Interval, **miR**: micro-Ribonucleic Acid, **TNF-α**: Tumor Necrosis Factor Alpha, **NF-κB**: Nuclear Factor Kappa B, **IL**: Interleukin.

#### 3.2.2. The Role of miRNAs as Therapeutic Targets

The role of miRNAs in IBD therapy has gained significance due to their involvement in pathological processes. As a result, it was concluded that monitoring the immune status according to the changes of miRNA signatures in the blood circulation and at the tissue level is an effective approach in IBD for the design of an individualized therapeutic strategy, having a predictive role in identifying the loss of the favorable response. Recently, miR-31 has shown promising results in order to reduce inflammation in experimental colitis models, indicating its potential therapeutic application [171]. Similarly, miR-223 has been found to alleviate symptoms such as occult bleeding and weight loss in DSS colitis models, suggesting its efficacy in improving clinical status [172]. By targeting dendritic cells, miR-29b possesses the ability to manage immune response [173]. Moreover, the therapeutic impact of miRNA-126 and miR-20a in pediatric CD patients treated with infliximab underscores their relevance in managing immune regulation and epithelial barrier function [174]. Inflamed tissues present elevated levels of miR-146a and miR-146b, which act as inhibitors of the NF-kB signaling pathway, presenting a mechanism to reduce inflammation. Moreover, miR-320a contributes to the healing of ulcers in the colonic mucosa, highlighting restorative properties [175]. The therapeutic implications of miR-29, particularly in reducing IL-23 expression by targeting the IL-12p40 subunit, validate its role as a biomarker for ustekinumab’s therapeutic efficacy [176] (Table 8).

## 4. Future Directions and Challenges

One significant implication is the advancement of personalized treatment strategies. By identifying specific genetic and epigenetic markers, clinicians could tailor therapies to individual patients, potentially improving treatment efficacy and reducing adverse effects. Future research may focus on the development of predictive models that utilize genetic and epigenetic data to identify individuals at high risk of developing IBD before clinical symptoms appear. Early detection could enable preventive measures and interventions, potentially altering the disease course and improving long-term outcomes. Moreover, understanding the genetic predisposition and early epigenetic changes provides insights into the initial triggers of IBD. The concept of multi-omics provides a comprehensive understanding of the molecular mechanisms underlying the diseases. Future perspectives include the development of integrated multi-omics platforms that create a holistic view of disease processes. These platforms could facilitate the discovery of novel biomarkers and therapeutic targets, enhancing the ability to diagnose, monitor, and treat IBD with unprecedented precision. The accuracy and potential of these biomarkers for in-depth exploration of genetic variation between populations must be verified in large cohorts of IBD patients in which datasets are collected longitudinally to ensure reproducibility and validation of the results regarding disease subtype classification and therapeutic intervention.

In recent years, the development of sequencing technology has provided new means of exploring the complex pathogenesis of IBD. Despite the progress achieved in describing the architecture of the genome and understanding the mechanisms related to the heredity of susceptible individuals, it is still claimed that they pose a minor contribution to the development or influence of the prognosis of these diseases since most of the risk alleles are extremely rare. Moreover, a real assessment of the impact of genotyping on therapeutic strategies is lacking, especially in patients who face the loss of favorable response over time. A major challenge associated with epigenetic studies is the heterogeneity of nonspecific IBD cells detected in blood or tissue samples, making data interpretation difficult due to the interference of different individual epigenetic characteristics. Despite the applications presented in this review, the usefulness of approaching genomic or epigenomic profiles in clinical practice is relatively limited due to the low accessibility for assessing complex analysis systems that, in addition, involve high costs in the context of obtaining small benefits. Moreover, there are a series of limitations of GWAS that refer, above all, to the linkage disequilibrium of the human genome in which different alleles could associate randomly and, subsequently, might be transmitted simultaneously.

However, when this imbalance is minimal, it is possible that the polymorphisms are not transmitted with the encoded gene and, as a result, they could not be detected by GWAS. On the other hand, it was found that the same gene variation might generate different clinical phenotypes through epigenetic changes or transcriptional and translational regulation. 

## 5. Conclusions

In the context of an incompletely elucidated pathogenesis, it was discovered that immunological, genetic, and environmental factors have an important contribution to the evolution of these diseases. Genomics and epigenetics explore the dimension of variation in the characteristics of these diseases, offering the advantage of understanding the cellular and molecular mechanisms that affect intestinal homeostasis for a much better prediction of the prognosis. However, the important perspective suggested by the studies included in this review is represented by the integration of genetic or epigenetic profiles in the field of precision medicine through the pretreatment detection of risk genes, which could improve the decision to apply a personalized strategy. The findings require an intensification of research aimed at epigenetic changes that are under selection pressure associated with the microbiome or intestinal inflammation, influencing the key pathways associated with the pathology of IBD.

## Figures and Tables

**Table 1:** Pattern of genes involved in altered epithelial barrier function in IBD.

Gene	Main Findings	References
***CDH1***	Responsible for E-cadherin synthesis, a transmembrane glycoprotein expressed in the intestinal epithelium, involved in cell adhesion and maintenance of mucosal barrier balance. Methylation of several CpGs at the CDH1 locus was increased in the inflamed ileal mucosa. Risk marker for gastric and colorectal cancer.	[28,29]
***GNA12***	Encodes the G protein Gα12, which causes destabilization of tight junctions and alteration of the intestinal barrier by phosphorylating zonulin ZO-1 and ZO-2. Anti-inflammatory factor that inhibits the excessive chemotactic migration of macrophages.	[30,31]
***PTPN2***	Negative regulator of pro-inflammatory cytokine signaling and counteracts IFN-γ-induced epithelial damage. Maintenance of intestinal barrier function. Loss of PTPN2 causes reduced autophagy and promotes the development of intestinal inflammation.	[32,33]
***HNF4-α***	Increases intestinal permeability by modulating the expression of RNF186 that alters barrier integrity at tight junctions. A protein-truncating R179X variant in RNF186 confers protection against UC.	[34,35]
***TRL-2***	Regulates intestinal permeability and tight junction translocation. Suppressing immune response and maintained mucosal integrity. Increased expression in the ileum of patients with active UC compared to inactive UC and healthy controls.	[36,37]
***IL-23R***	Inhibition of IL-23 or IL-23R function reduced intestinal mucosal inflammation and confers protection against IBD. Stimulation of IL-23R inhibits Th17 cells by IL-22 and IL 17-mediated reduction of commensal microbiota and maintains barrier function.	[38,39]
***IL-17R***	Interacts with IL-17R receptor to stimulate inflammatory reactions through the action of mitogen-activated protein kinase MAPK, NF-Κb, and JAK/PI3K signaling. Inhibiting IL-17RA reduces inflammation and restores intestinal barrier function.	[40,41]

***CDH1***: Cadherin 1, ***GNA12***: G Protein Subunit Alpha 12, ***PTPN2***: Protein Tyrosine Phosphatase Non-Receptor Type 2, ***RNF186***: Ring Finger Protein 186, ***IL-23R***: Interleukin-23 Receptor, ***IL-17R***: Interleukin-17 Receptor, **CpGs**: Cytosine-phosphate-Guanine dinucleotides, HNF4-α: Hepatocyte nuclear factor 4α, ***IFN***: Interferon, ***MAPK***: Mitogen-Activated Protein Kinase, NF-κB: nuclear factor-kappa B, JAK: Janus kinase, PI3K: phosphatidylinostitol 3-kinase.

**Table 2:** Genes involved in IBD in different populations.

Gene	Population Group	Disease	Main Findings	References
***NUDT15***	Asian	IBD	Anticipates thiopurine-induced myelosuppression. Predicts development of stenosis or fistulas.	[42]
***TNFSF15***	Asian	CD	Predictive for the development of complications (strictures, penetrating disease, and perianal fistula).	[43]
***FUT2***	Asian	IBD	The FUT2 loss-of-function mutation alters the gut microbiota by decreasing the abundance of adherent bacteria and stimulating CD8+ and Th17 cells that produce inflammatory lesions. Found in about 20–60% of Asian and Caucasian populations.	[44,45]
HLA-DQA1*05	European	CD	Increased risk of immunogenicity to anti-TNF agents. Carried by approximately 40% of Europeans.	[46,47]
HLA-DRB1*0103	European	CD	Associated with extra-intestinal manifestation.	[48]
HLA-DRB1*1501	European	CD	Has been involved in extensive ulcerative colitis with severe evolution. Encountered in 6–25% of people of European ancestry.	[49]
HLA-DRB1*07	European and African American	CD	Frequently associated with ileal disease. This allele varies between 5% and 29% in Europeans and North Americans but is less than 1% in Japanese.	[24]
***NOD2 (CARD15)***	European and African American	CD	Regulation of inflammation and cell apoptosis. This gene is linked to early onset, severe evolution, and stenotic complications requiring surgical interventions.	[50]
HLA-DQA1*05	European and African American	IBD	Associated with immunogenicity and loss of response to anti-TNF agents.	[51]
***TPMT***	European	IBD	Anticipates thiopurine-induced myelosuppression. About 25% of patients with European ancestry carry specific variants.	[52]
***ATG16L1***	European and African American	CD	Destabilizes caspase-3 in Paneth cells. Reduces autophagy and intracellular bacterial clearance. Stimulates the release of proinflammatory cytokines.	[53]

***NUDT15***: Nudix Hydrolase 15, ***TNFSF15***: Tumor Necrosis Factor (Ligand) Superfamily, Member 15, ***FUT2***: Fucosyltransferase 2, HLA-DQA1*05: Human Leukocyte Antigen DQ Alpha 1*05, HLA-DRB1*0103: Human Leukocyte Antigen DR Beta 1*0103, HLA-DRB1*1501: Human Leukocyte Antigen DR Beta 1*1501, HLA-DRB1*07: Human Leukocyte Antigen DR Beta 1*07, ***NOD2 (CARD15)***: Nucleotide-Binding Oligomerization Domain Containing 2 (Caspase Recruitment Domain Family, Member 15), ***TPMT***: Thiopurine S-Methyltransferase, ***ATG16L1***: Autophagy Related 16 Like 1, ***IBD***: Inflammatory Bowel Disease, **CD**: Crohn’s Disease, ***CD8+***: CD8-positive T cells, ***Th17***: T helper 17 cells, ***anti-TNF***: Anti-Tumor Necrosis Factor.

**Table 3:** Single-nucleotide polymorphism and the response to anti-TNF therapy.

Biologic Treatment	Disease	Techniques Used/Samples	Studied Genes	Treatment Effectiveness	References
**IFX** **ADA**	IBD pediatric cohort	RT-PCR Serum	rs2097432 HLA-DQA1*05	Decreased response: - rs2097432 CD vs. UC (HR: 2.23; 95% CI: 1.36–3.66; *p* = 0.001); - IFX vs. ADA (HR: 1.70; CI 95%:1.02–2.77; *p* = 0.043);	[87]
**IFX**			rs2395185 *HLA-DRB9*	- rs2395185 CD vs. UC (HR: 2.07; 95%CI: 1.27–3.37; *p* = 0.004); - IFX vs. ADA (HR: 1.74; CI 95%: 1.05–2.87; *p* = 0.03).	
**IFX** **ADA**	CD	RT-PCR Colonic tissue	rs1052571 rs4645978 *CASP9*	Increased response: - rs1052571 (OR: 2.40; 95% CI: 1.12–5.34; *p* = 0.0224);	[88]
				- rs4645978 (OR: 3.00; 95% CI: 1.17–7.68; *p* = 0.00117).	
**IFX**	CD	MassArray Analyzer System Serum	rs442905 *C1orf106*	Decreased IFX levels in GA carriers (*β* = −0.949; *p* = 0.025).	[89]
			rs7587051 *ATG16L1*	Decreased IFX levels in GG genotype.	
			rs3213448 *IL1RN*	Increased IFX levels in GA carriers (OR: 2.14; 95% CI: 0.99–4.77).	
**IFX**	IBD pediatric cohort	RT-PCR Serum	rs5030728 TLR4	Decreased IFX levels in GG’ vs. GA + AA (OR: 3.434; 95% CI: 1.35–8.71; *p* = 0.020).	[90]
**IFX**	CD	RT-PCR Serum	rs3024505 IL-10	Increased IFX levels.	[91]
**IFX**	CD	RT-PCR Serum	rs10489629 *IL-23R*	Protective effect against failure to respond to IFX; the lack of response to IFX was 75% lower.	[92]
			rs10929587 *ADAM17* rs3794271 *SLCO1C1*	Increased risk of CD-related surgery (OR = 2.8; 95% CI = 1.1–7.3; *p* = 0.025).	
**IFX** **ADA**	CD	RT-PCR Serum	rs11209026 *IL-23R*	Increased risk of CD-related surgery.	[93]
**IFX**	IBD	RT-PCR Serum	rs5030728 *TLR4*	Increased response G allele in rs5030728 in CD (OR: 2.23; 95% CI: 1.24–4.01).	[86]
			rs10499563 IL-6	Decreased response C allele in rs10499563 (OR: 1.31; 95% CI: 0.99–1.71).	
**IFX**	CD	RT-PCR Serum	rs766748 IL-17	Increased response: G/G genotype (OR: 5.123; 95% CI: 1.261–27.77, *p* = 0.0213).	[94]
**IFX**	CD	RT-PCR Serum	rs1799724 TNF-α	Decreased response 857T allele higher production of TNF-α in the intestine.	[95]
**ADA**	CD	RT-PCR Serum	rs10210302 *ATG16L1*	Increased response C/T and T/T genotype (OR: 9.44; 95% CI: 2.49–35.83).	[96]

**IFX**: Infliximab, **ADA**: Adalimumab, **IBD**: Inflammatory Bowel Disease, **RT-PCR**: Reverse Transcription Polymerase Chain Reaction, **HLA**: Human Leukocyte Antigen, **CASP**: Caspase, **IL**: Interleukin, **TNF**: Tumor Necrosis Factor, **TLR**: Toll-like Receptor, **ADAM**: A Disintegrin and Metalloprotease, **SLCO**: Solute Carrier Organic Anion Transporter, **CI**: Confidence Interval, **OR**: Odds Ratio, **HR**: Hazard Ratio, **UC**: Ulcerative Colitis, **CD**: Crohn’s Disease, **DQA**: HLA-DQ Alpha, **rs**: Reference SNP (Single-Nucleotide Polymorphism), **DRB**: HLA-DR Beta, **C1orf106**: Chromosome 1 Open Reading Frame 106, **ATG16L1**: Autophagy Related 16 Like 1, **IL1RN**: Interleukin 1 Receptor Antagonist, **GA**: Guanine-Adenine (a heterozygous genotype), **GG**: Guanine-Guanine (a homozygous genotype), **G/G**: Guanine-Guanine (a homozygous genotype), **857T**: Thymine at position 857, **C/T**: Cytosine–Thymine (a heterozygous genotype), **T/T**: Thymine–Thymine (a homozygous genotype).

**Table 4:** The impact of various epigenetics mechanisms regarding the pathogenesis of IBD.

Biomarker	Disease	Sample	Main Findings	References
**DNA methylation analysis**				
***TAP1*** ***TESPA1*** ***RPTOR***	IBD	Blood	Hypomethylation model with 3 DMPs was correlated with treatment escalation to biological agents or surgery in IBD (HR 5.19; CI 95%: 2.14–12.56).	[110]
***RPS6KA2*** ***VMP1*** ***TNSF10***	CD UC	Blood	The paired hypomethylation probe biomarkers RPS6KA2/VMP1 and RPS6KA2/TNFSF10 accurately discriminate between controls and CD (AUC = 0.84/0.81) or UC (AUC = 0.73/0.71); VMP1/microRNA-21 methylation related with IBD susceptibility.	[111]
***FKBP5***	CD UC	Blood	The polymorphism (rs4713916) in the putative promoter region of FKBP5—associated with glucocorticoid resistance only to CD (genotype GC vs. GA/AA, OR: 3.81; CI 95%: 1.66–8.75).	[112]
***BCL3***	UC CD	Colonic tissue	Influences the severity of intestinal ulcerations. Hypomethylation of BCL3—strongly elevated in active UC and active or inactive CD.	[113,114]
***EYA4*** ***SLIT2*** ***FLI1*** ***USP44*** ***SND1***	IBD	Colonic tissue	Early detection of colorectal cancer and dysplastic lesions in IBD patients compared to healthy controls (92% vs. 57%; OR: 8.63; *p* = 0.001).	[106]
***CND1*** ***OL4A2*** ***HDAC2*** ***GLI2*** ***AXIN2*** ***ABL1***	UC	Colonic tissue	Strong concordance of methylation alterations (hypermethylation and hypomethylation) in the cancer cells and the UC samples (OR: 22.15; CI 95%: 15.48–31.56, *p* < 0.001).	[115]
***TGFβ1***	CD UC	Blood	Panels of methylation marks enable accurate diagnosis of CD (14 *TGFβ1 +* 4 *IL-6* CpG sites, AUC = 0.95), UC (9 TGFβ1 CpG sites; AUC = 0.99 and 4 IL-6 CpG sites, AUC = 0.89), and discrimination between CD and UC (3 TGFβ1 CpG sites, AUC = 0.81).	[116]
**Histone-modifying enzymes**				
***HDAC***	IBD	Colonic tissue	HDAC inhibitors influences intestinal homeostasis by maintaining the integrity of the epithelial barrier. They modulate the expression of tight junction proteins (claudin-1, claudin-2, and occludin) and increase the synthesis of TGF-β1 and IL-8 to stimulate the healing of inflammatory lesions.	[117]
***Setd2***	Experimental model colitis on mice	Cell culture Colonic tissue	Deletion of Setd2 stimulates the generation of NKp46 + ILC3 with enhanced cytotoxic signatures and tumor suppressive capacity.	[118]
			Modulates the expression of Treg cells that inhibit the development of intestinal inflammatory lesions.	[119]
***HDAC3***	Experimental model colitis on mice	Cell culture Colonic tissue	Influences the intestinal immunity (reduces the number of CD4+ T cells and stimulates the major histocompatibility complex class II (MHC II), with a role in the control of inflammatory lesions.	[120]
			Therapeutic target: inhibit NF-κB signaling, downregulation of tight-junction proteins and stimulation of intraepithelial lymphocytes during infection.	[121]
**HDAC1** **HDAC5**	Experimental model colitis on mice	Cell culture Colonic tissue	Impact on intestinal permeability and reduce inflammation by promoting mucosal colonization with *Enterobacteriaceae*.	[122]
			Therapeutic target HDAC1—negative regulator of STAT signaling, NF-κB signaling, and acute phase response; stimulation of IL-1β-dependent cytokine production.	[121]
**SETDB1**	Experimental model colitis on mice	Blood Feces Colonic tissue	It acts on endogenous retroviruses to inhibit DNA damage. The deletion is accompanied by damage to the integrity of the intestinal barrier, epithelial differentiation disorders, and exacerbation of inflammatory lesions with a severe prognosis.	[123]
**SETDB1**	IBD	Cell culture Colonic tissue	SETDB1 deletion causes intestinal stem cell instability, with the release of endogenous retroviruses and necroptosis dependent on ZBP1 that influences inflammatory lesions.	[124]
**lncRNAs**				
**KIF9-AS1**	CD UC	Colonic tissue Blood	Expression levels were significantly higher in patients with UC and CD compared with the healthy controls (AUC = 0.872 and 0.811, respectively, *p* < 0.0001).	[125]
**LINC01272**			Expression levels were significantly higher in patients with UC and CD compared with the healthy controls (AUC = 0.777 and 0.887, respectively, *p* < 0.001).	
**DIO3OS**			Expression levels were significantly lower in patients with UC and CD compared with the healthy controls (AUC = 0.653 and 0.794, respectively, *p* < 0.0001).	
**ANRIL**	CD	Colonic tissue	The low expression in the intestinal mucosa differentiates CD from healthy controls (AUC = 0.803, 95% CI: 0.733–0.874, sensitivity = 0.861, specificity = 0.642) and the active remission stage of the disease (AUC = 0.839, 95% CI: 0.760–0.918, sensitivity = 0.857, specificity = 0.712).	[126]
			An increased level is associated with favorable response to infliximab.	
***RP11–679B19.1***	CD	Ileal tissue	Strong association between increased expression and the WWOX gene in patients with recurrent fibrostenotic CD carrying the risk allele A (OR = 4.13, *p* < 0.01).	[127]

**TAP1**: Transporter 1, ATP Binding Cassette Subfamily B Member, **TESPA1**: Thymocyte Expressed, Positive Selection Associated 1, **RPTOR**: Regulatory Associated Protein of MTOR Complex 1, **RPS6KA2**: Ribosomal Protein S6 Kinase A2, **VMP1**: Vacuole Membrane Protein 1, **TNFSF10**: Tumor Necrosis Factor Superfamily Member 10, **FKBP5**: FK506 Binding Protein 5, **BCL3**: B-Cell Lymphoma 3, **EYA4**: Eyes Absent Homolog 4, **SLIT2**: Slit Guidance Ligand 2, **FLI1**: Friend Leukemia Integration 1 Transcription Factor, **USP44**: Ubiquitin Specific Peptidase 44, **SND1**: Staphylococcal Nuclease and Tudor Domain Containing 1, **CND1**: Cyclin D1, **OL4A2**: Oligophrenin 4, Autosomal 2, **HDAC**: Histone Deacetylase, **GLI2**: GLI Family Zinc Finger 2, **AXIN2**: Axis Inhibition Protein 2, **ABL1**: ABL Proto-Oncogene 1, Non-Receptor Tyrosine Kinase, **TGFβ1**: Transforming Growth Factor Beta 1, **Setd2**: SET Domain Containing 2, **SETDB1**: SET Domain Bifurcated 1, **KIF9-AS1**: Kinesin Family Member 9 Antisense RNA 1, **LINC01272**: Long Intergenic Non-Protein Coding RNA 1272, **DIO3OS**: Dio3 Opposite Strand/Antisense RNA, **ANRIL**: Antisense Non-Coding RNA in the INK4 Locus, **RP11–679B19.1**: Long Non-Coding RNA associated with WWOX Gene, **IBD**: Inflammatory Bowel Disease, **UC**: Ulcerative Colitis, **CD**: Crohn’s Disease, **DMPs**: Differentially Methylated Positions, **RPS6KA2**: Ribosomal Protein S6 Kinase A2, **TNFSF10**: Tumor Necrosis Factor Superfamily Member 1, **rs4713916**: Reference SNP (Single-Nucleotide Polymorphism) 4713916, **GC vs. GA/AA**: Genotypes involving combinations of Guanine (G), Cytosine (C), Adenine (A), GC (heterozygous), GA/AA (heterozygous and homozygous for A, respectively), **CpG**: Cytosine-phosphate-Guanine, **IL**: Interleukin, **ZBP1**: Z-DNA-binding protein 1, **MHC II**: Major Histocompatibility Complex Class II, **NKp46**: Natural Killer Cell P46-Related Protein, **ILC3**: Group 3 Innate Lymphoid Cells, **Treg cells**: Regulatory T Cells, **NF-κB**: Nuclear Factor Kappa-Light-Chain-Enhancer of Activated B Cells, **STAT**: Signal Transducer and Activator of Transcription, **IL-1β**: Interleukin 1 Beta, **AUC**: Area Under the Curve, **HR**: Hazard Ratio, **OR**: Odds Ratio, **CI**: Confidence Interval.

**Table 5:** The role of lncRNA in IBD pathogenesis.

lncRNA	Main Findings	References
**ANRIL**	Low expression in intestinal mucosa; increased secretion of proinflammatory cytokines; differentiates between active and remission stages of CD.	[126]
**RP11–679B19.1**	Strong association with WWOX gene in recurrent stenotic CD (OR = 4.13, *p* < 0.01).	[127]
**BC012900**	Elevated in patients with active UC; its overexpression in intestinal epithelium increases susceptibility to apoptosis.	[133]
**HNF4A-AS1**	Related to severe mucosal ulcerations in pediatric patients; affects expression of HNF4A gene, regulating epithelial barrier function.	[134]
**LINC01272**	Expressed in monocytes and neutrophils in intestinal epithelium; related to severe mucosal ulcerations; increased level correlated with fecal calprotectin (r = 0.9, *p* < 0.01).	[134]
**DIO3OS**	Biomarker with high performance to differentiate IBD patients from healthy controls.	[125]
**KIF9-AS1**	Elevated levels in UC and CD patients; potential as a biomarker for disease activity and progression.	[125]

**ANRIL**: Antisense Noncoding RNA in the INK4 Locus; **CD**: Crohn’s Disease; **UC**: Ulcerative Colitis; **IBD**: Inflammatory Bowel Disease; **HNF4A**: Hepatocyte Nuclear Factor 4 Alpha; **LINC01272**: Long Intergenic Non-Protein Coding RNA 1272, **DIO3OS**: DIO3 Opposite Strand/Antisense RNA, **KIF9-AS1**: Kinesin Family Member 9–Antisense RNA 1, **WWOX**: WW domain-containing oxidoreductase, **lncRNA**: long non-coding RNA.

**Table 6:** Types of miRNAs signatures in inflammatory bowel disease.

miRNAs	Target	Main Findings	References
**Intestinal inflammation**			
**miR-223**	NLRP3	Inhibits NLRP3, which decreases the release of IL-1β and favors the healing process.	[141]
**miR-23a** **miR-155**	Neutrophils	Reduces the infiltration of the intestinal tissue with activated neutrophils; prevents further injuries and the development of neoplasia.	[142]
**miR-155**	Macrophages	Release at the intestinal level induces the differentiation of M1 macrophages, which increases the inflammatory lesions.	[143]
**miR-21**	TNF-α MIP-2	Stimulates the secretion of TNF-α and macrophage inflammatory protein 2 (MIP-2), which intensifies inflammatory lesions.	[144]
**miR-10a**	NOD2 IL-12/ IL-23p40	Downregulates the expression of NOD2 and IL-12/IL-23p40 in the inflamed mucosa and suppresses T helper (Th)1 and Th17 cell responses.	[145]
**Epithelial barrier function**			
**miR-200b**	claudin-1 zonulin-1	Inhibits TNF-α and IL-8 and prevents the increase in paracellular permeability by redistributing proteins of intercellular tight junctions (claudin-1 and zonulin-1).	[146]
**miR-675**	zonulin-1 cadherin E	Increases intestinal permeability by altering zonulin 1 and E-cadherin at the level of intercellular tight junctions.	[132]
**miR-122a**	EGFR	Increases the expression of zonulin-1, which is correlated with the dysfunction of the intestinal epithelium.	[147]
**miR-21**	PTEN p-Ak pathway	Hyperproduction suppresses tensin (PTEN) expression and increases phospho-Akt (p-Akt) levels to improve paracellular permeability.	[148]
**miR-191a**	zonulin-1	TNF-α stimulation increased miR-191a expression, leading to the decline in zonulin-1 and increased intestinal permeability.	[149]
**Autophagy**			
**miR-665**	XBP1 ORMDL3	Promotes apoptosis by repressing the endoplasmic reticulum stress components XBP1 and ORMDL3.	[150]
**miR-20a**	ECN1 ATG16L1 SQSTM1	High levels were associated with downregulation of ECN1, ATG16L1, and SQSTM1, which inhibit autophagy.	[151]
**miR-106b** **miR-93**	PTEN ATG16L1 Akt pathway	Suppression of ATG16L1 expression and PTEN activity inhibits autophagy and stimulates the development of neoplastic lesions.	[152]
**miR-16**	Bcl-2	Downregulates the expression of Bcl-2 to disrupt colonic epithelium and inhibit autophagy.	[153]
**miR-143**	ATG2B	Inhibits autophagy and increases inflammatory responses.	[154]
**miR-132** **miR-223**	FOXO3	Downregulate FOXO3, which enhances NF-κB signaling and inhibits autophagy.	[155]

**NLRP3**: NLR Family Pyrin Domain Containing 3, **IL**: Interleukin, **TNF-α**: Tumor Necrosis Factor Alpha, **MIP-2**: Macrophage Inflammatory Protein 2, **NOD2**: Nucleotide-binding Oligomerization Domain-containing Protein 2, **Th1**: T-helper 1, **Th17**: T-helper 17, **EGFR**: Epidermal Growth Factor Receptor, **PTEN**: Phosphatase and Tensin Homolog, **p-Akt**: Phospho-Protein Kinase B, **XBP1**: X-box Binding Protein 1, **ORMDL3**: Orosomucoid-like Protein 3, **ECN1**: Echinoderm microtubule-associated protein-like, **ATG16L1**: Autophagy Related 16 Like 1, **SQSTM1**: Sequestosome 1, **Akt**: Protein Kinase B, **Bcl-2**: B-cell Lymphoma 2, **ATG2B**: Autophagy Related 2B, **FOXO3a**: Forkhead Box O3, **NF-κB**: Nuclear Factor Kappa-light-chain-enhancer of Activated B Cells.

**Table 8:** Impact of miRNAs on therapeutic response in IBD.

miRNAs	Role/Therapy	References
**miR-31**	Significant reduction in colonic inflammation after capsule administration in mice with experimental colitis.	[171]
**miR-223**	Decrease in occult bleeding, weight loss, and edema after intracolonic application of a nanoparticle emulsion in mice with DSS colitis.	[141]
**miR-29b**	Reduction in inflammatory response and inhibition of immune response through subcutaneous injection in mice with DSS colitis.	[172]
**miR-126,** **miR-20a**	Significantly decreased levels in serum and feces after infliximab administration in pediatric CD patients.	[173]
**miR-126, miR-146a,** **miR-146b, miR-320a, let-7c, miR-636, miR-193b**	Clinical and therapeutic response in pediatric patients with IBD is associated with overexpression of miR-636 and miR-193b, and decreased levels of miR-320a, miR-126, let-7c, miR-146a, and miR-146b. miR-320a involved in ulcer healing in intestinal mucosa.	[174]
**miR-29**	Reduces IL-23 expression by targeting the IL-12p40 subunit, considered a parameter of ustekinumab therapy efficacy.	[175]
**miR-3934, miR-100, miR-718, miR-193b, miR-3150a-5p, miR-1260b, miR-938, miR-518b, miR-1468.**	Pharmacodynamic biomarkers used to stratify responders versus nonresponders to infliximab therapy (84% accuracy, AUC = 0.82).	[176]

**IBD**: Inflammatory Bowel Disease, **CD**: Crohn’s Disease, **DSS**: Dextran Sodium Sulfate, **IL**: Interleukin, **AUC**: Area Under the Curve, **miR**: micro-Ribonucleic Acid.

## Data Availability

Not applicable.
